# Salt wedges and trapped brines of low-latitude endoreic saline lakes as potential modulators of GHG emission

**DOI:** 10.1038/s41598-023-48148-8

**Published:** 2023-11-30

**Authors:** Elisabeth Gibert-Brunet, Alina Tudryn, Ting Kong, Piotr Tucholka, Seyed-Hani Motavalli-Anbaran, Christelle Marlin, Aurélie Noret, Mohammad Lankarani, Hesam Ahmady-Birgani, Gilda Karimi

**Affiliations:** 1 CNRS, UMR 8148-GEOPS, University of Paris-Saclay, 91405 Orsay, France; 2 Faculty of Geology, Warsaw University, 02-089 Warsaw, Poland; 3 Institute of Geophysics, University of Tehran, Tehran, Iran; 4 School of Geology, University-College of Science, University of Tehran, Tehran, Iran; 5 Faculty of Natural Resources, Urmia University, Urmia, Iran; 6 Faculty of Biological Sciences, Kharazmi University, Tehran, Iran

**Keywords:** Environmental sciences, Hydrology, Limnology

## Abstract

Large salt lakes are long-term witnesses to climatic conditions and land use in their basins. The majority are experiencing a drastic drop in water levels due to climate change and human impact. Endoreic Lake Urmia (NW Iran), the sixth largest salt lake worldwide, is a striking example of this decline. Quantification of the relative contributions of natural variability and human impact on the lake's water supply is therefore essential. Here we present isotopic and radiocarbon analyses of surface and groundwater from the Shahr Chay River catchment, entering Lake Urmia on its western shore, and radiocarbon dating of a sedimentary core. Lake Urmia behaves like a large saltwater wedge almost entirely fed by the river and shallow groundwater. This leads to trapping of residual brines and formation of CH_4_ and secondary CO_2_ greenhouse gases, impacting sediment geochemical records and corresponding time scales for paleoenvironmental reconstructions. We conclude that (1) salt lakes functioning like a saline wedge, allowing organic matter oxidation, could contribute to increasing methane sources or reducing carbon sinks globally, and (2) endoreic basins worldwide need to be monitored before aridification-related salinization leads to the establishment of a saline wedge precluding any possibility of return to an equilibrium state.

## Introduction

The overall decrease in surface waterbodies in recent decades, especially in semi-arid and arid regions, is a visible sign of decreasing aquifer recharge. This may reflect either rainfall modification or evapotranspiration indices linked to climate change, or growing impact of anthropogenic activities, through misuse of water, intensive pumping, or population increase in areas already under pressure for freshwater resources^1,2^. In salt lake basins, groundwater discharge into lakes is of utmost importance in supporting the hydro-ecological system by supplying water and nutrients, mitigating water salinity, and limiting temperature amplitudes^3,4^. Strongly dependent on groundwater management, the sustainability of these basins is often studied by modelling, due to their complex structures, the various processes they undergo, and lack of systematic observation data on the surface- and groundwater compartments, lacustrine deposits, and their interconnections^5^. In addition to studies of present-day surface water-groundwater interactions, accurate comparison of paleoclimate records^6–8^ is essential to understand the timing, causality and mechanisms of environmental changes. Regarding lake sediments, the major challenges are to identify the processes that may have distorted radiocarbon chronology, nowadays or in the past, and to define a reliable time-scale of environmental phases. These issues are particularly relevant in the case of complex hydrogeological basins impacted by tectonics, volcanism or hydrogeochemical processes, as in saline environments.

**Figure 1:**
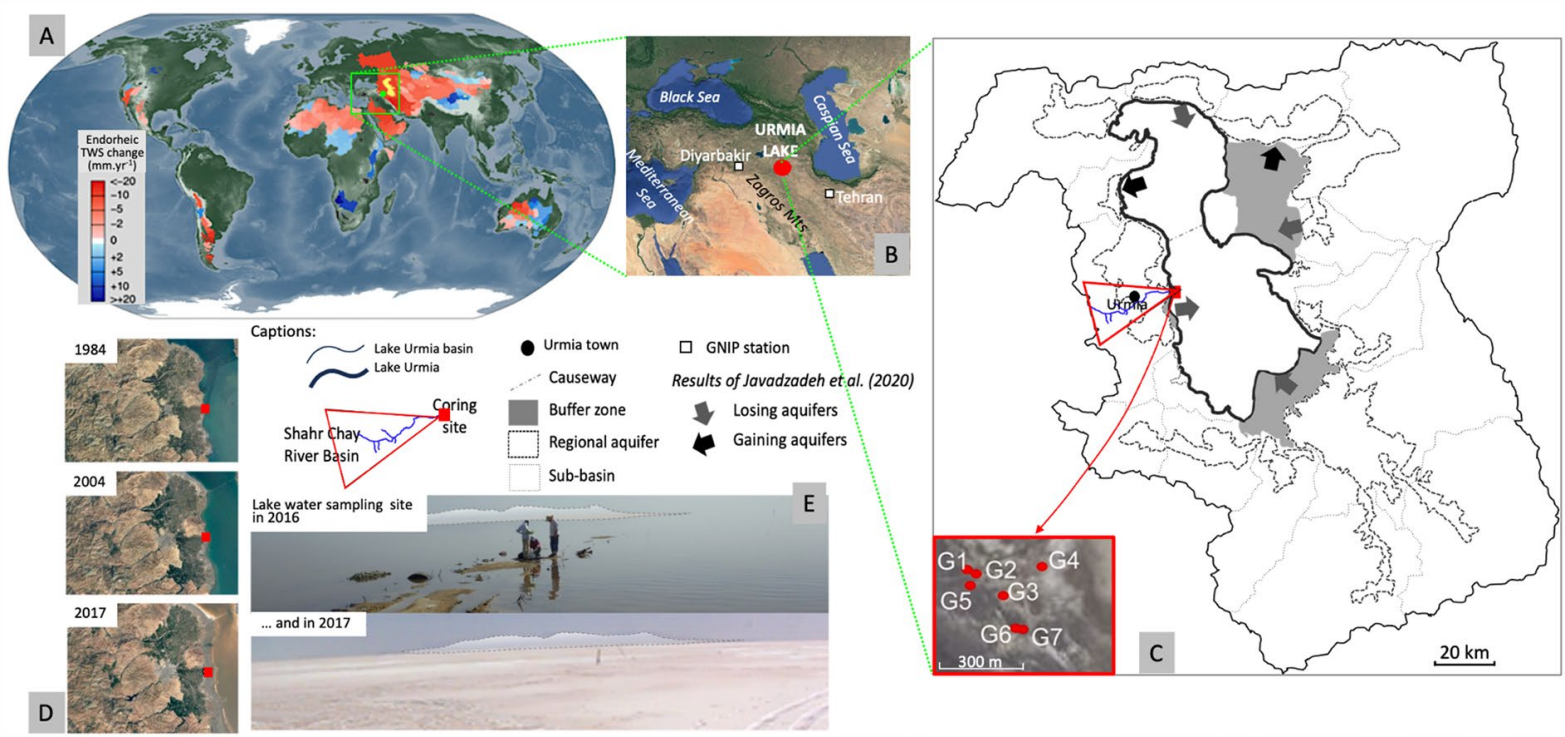
Location of Lake Urmia (Iran) (**A**) on the global map of the major endoreic systems at the global scale^2^, (**B**) within the regional context of the Middle East region; (**C**) Lake Urmia basin with mention of showing the sub-basins separated by the causeway, regional aquifers, buffer zones, and the location of the 7 cored sedimentary sequences (G1 to G7) at the Shahr Chay River mouth^21^; (**D**) Lake Urmia water evolution at coring site between 1984 and 2017; E. Photos of the lake surface water sampling site (close to G4 coring) during the two field missions in 2016 and in 2017.

On a global scale, the decline of these large waterbodies presents huge problems, such as those faced in the Iranian Lake Urmia basin, which experienced a drop of more than 8 m in water level over the last 20 years^2,9–11^ (Fig. 1). This decline has been attributed, albeit controversially, to the 10% reduction in rainfall amount and, for more than half the groundwater losses, to anthropogenic impacts^12^, due to too many dams being built on rivers^13^, excessive pumping, and intensive agriculture without efficient irrigation techniques^14^. This has led to soil salinization and increase in dust storms, resulting in lower agricultural production, health problems and mass emigration of rural communities^10–12^. Unfortunately, these consequences are likely to worsen, as recent modelling of Lake Urmia basin predicts an increase in the frequency of hot, dry months from 4.7% to 24.0% over the period 2060–2080^15^.

In 2013, the Iranian administration launched the Lake Urmia Restoration Program (ULRP) with the objectives of understanding the origin of Lake Urmia’s drying-up and remedying this, as well as restoring the former lake level by assessing and managing water resources and ecological services^1,16,17^. Despite the obvious groundwater overexploitation, the respective roles of natural and anthropogenic factors influencing the lake’s hydrology have not yet been quantified and the dynamics of this particular eco-hydrosystem has been totally neglected in the proposed restoration efforts^1^. The lack of data on present and past environments limits understanding of the processes that determine the current state of the lake system and its future evolution, thereby hampering the development of this integrated program of resource management and ecological restoration.

The comparison of satellite data, field monitoring and land-surface models indicated that over half the groundwater loss can be attributed to human impacts^13^. Recent hydrochemical data on plain aquifers at the northeastern lake shore indicate a higher salinization due to deforestation, evaporated return flows from irrigation, and/or rock dissolution in the catchment and as a conclusion, no strong interaction between the lake water and the surrounding aquifers^14,18^. On the opposite, hydrological modelling based on various scenarios of water use in the Lake Urmia catchment area or on specific methodologies associating Darcy's law and inverse modeling of lake bed’s hydraulic conductivity, have highlighted that the surface water baseflow feeding the lake is almost essentially supported by shallow groundwater extending over the entire Lake Urmia catchment area^5,13,15,16,20^. These modeling point also that (1) the lack or underestimation of field data as well as the inadequate consideration of socio-economic parameters do not yet allow fully convincing results to be achieved by actions undertaken to reverse the evolution of the lake^20^, and (2) only the “cessation-of-any-withdrawal-of-water-from-Lake-Urmia” could achieve the objectives of the lake restoration program^5,15,16,19^.

Part of the controversy over Lake Urmia's decline stems from modelling potentially biased by a sub-critical geochemical data set insufficient to constrain water flows. In order to verify the hypotheses of connection, or not, between groundwater and the lake, and to confirm or refute existing models, we undertook to produce quantitative hydrogeochemical data on the western basin of the lake. We thus present here the first isotopic data on δ^18^O, δ^13^C_TDIC_ [Total Dissolved Inorganic Carbon], and δ^2^H, as well as the first ^14^C_TDIC_ activity data from 16 surface water and groundwater samples from Lake Urmia’s western basin drawn from a unique set of samples collected in 2016 and 2017, encompassing water samples, sedimentary sequences and geological samples collected in the recently dried-out western part of the lake in the Shahr Chay river basin (Fig. 1)^21–23^. Our isotopic study will aim at deciphering the specific lake evolution in tight connection with both the lake modern reference system and its paleoenvironmental history and the data will be made available to decision-makers for inclusion in the restoration project. This will also enable us to develop a conceptual model of the interactions between surface- and groundwater, permitting validation of the radiocarbon chronology established on cored sediments^21–23^ for global paleoenvironmental reconstructions during the Late Quaternary.

## Results

### Lake Urmia reference hydrosystem: modern water and old brines

**Figure 2:**
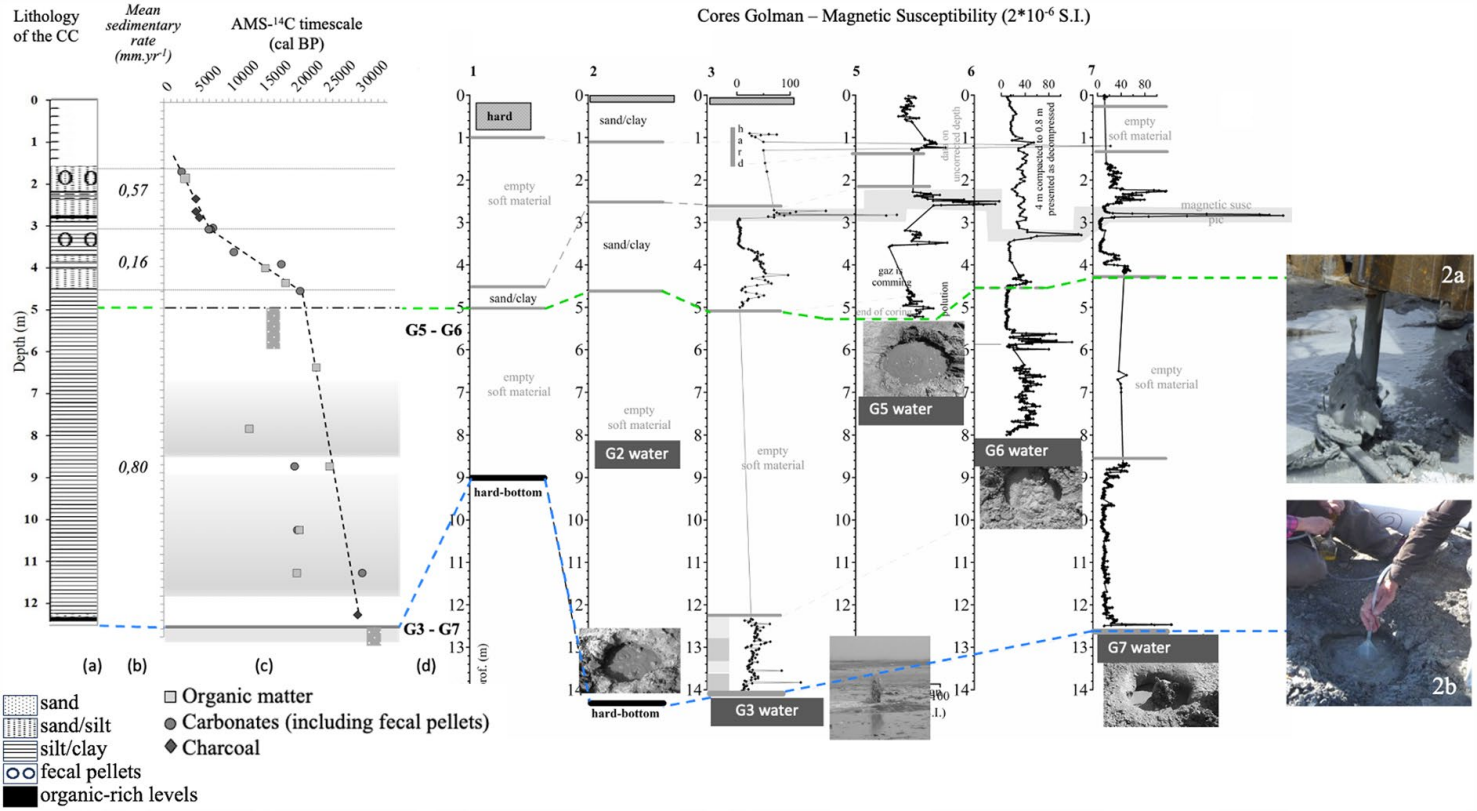
Lithology (**a**) and ^14^C time-scale with (**b**) sedimentary rate, and (**c**) AMS ^14^C datings^21,22^ of the Composite core (C-core) reconstructed based on the 7 boreholes (numbered 1 to 7) drilled at the Sahar Chay River mouth during the 2016 and 2017 field works. The muddy waters from coring wells (**d**) are located within the corresponding empty sections.

Existing scientific literature on Lake Urmia hydrogeology stresses, very often based on modelling, that the lake is mainly fed by rivers, and that flows between the lake and groundwater are limited or non-existent^16–18,20,24,25^. Our study highlights a clear specificity linked to the basin salinization: our sediment cores show a succession of well-defined lacustrine deposits alternating with mud-filled "empty" sections characterized by outgassing of varying intensity (Fig. 2). The two deepest mudflows G3 and G7 were brought to the surface by artesian extraction, which was accompanied by intense outgassing of H_2_S and petroleum vapour, strongly suggesting the presence of methane. The G3 liquid sludge was sampled under secure conditions (casing avoiding any contamination)^21^ and like all other samples, analyzed for ^18^O, ^2^H and ^13^C_TDIC_ contents, as well as for ^14^C_TDIC_ activities.

**Figure 3:**
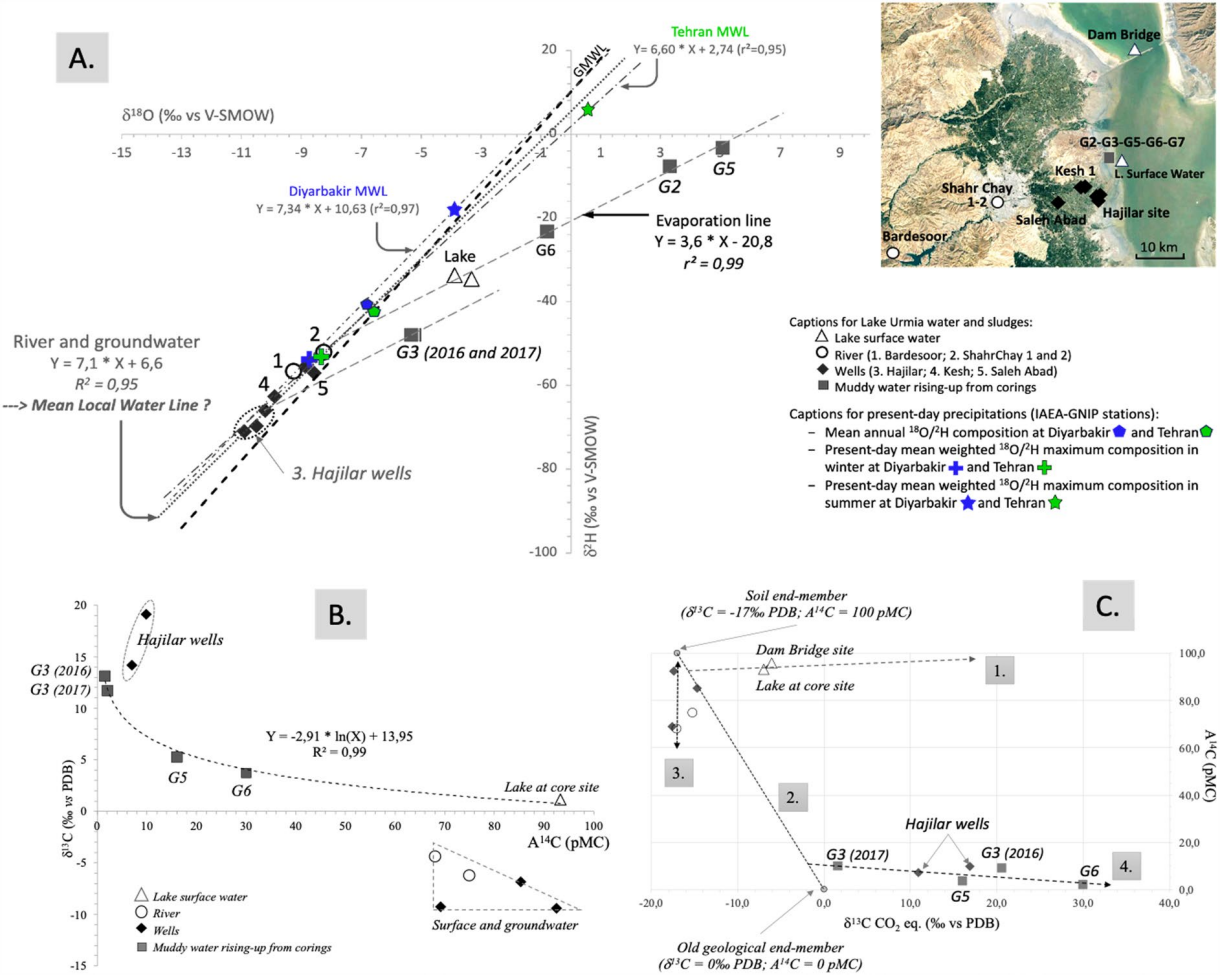
(**A**) Stable isotope contents of surface and groundwater in the Shahr Chay River basin and of muddy water retrieved form Lake Urmia corings. The mean stable isotope compositions of rainfalls at the Diyarbakir (Turkey) and Tehran (Iran) stations are defined according to GNIP data (GNIP Database, IEAE/WMO, 2023; average weighted annual summer and winter means); (**B**) δ^13^C_TDIC_ content versus ^14^C activity diagram for surface and groundwater from the Shahr Chay River basin and for Lake Urmia surface water; (**C**) Diagram δ^13^C_PCO2_ vs A^14^C and geochemical processes determined in the Lake Urmia basin.

Surface water and groundwater in the basin showed isotopic values ranging from − 10.9 to + 3.3 ‰ and from − 71.0 to − 3.3 ‰ for δ^18^O and δ^2^H respectively (Table 1, Methodology for units). In a δ^2^H vs δ^18^O diagram, these data defined a local meteoric water line (LMWL) with an equation intermediate between those of the two closest GNIP stations in Diyarbakir (Turkey) and Tehran (Iran; IAEA/WMO, 2022; Figs. 1 and 3A). The present-day isotopic value of the Shahr Chay River is very close to that of the winter precipitation recorded by these two stations. Without neglecting direct lake recharge, we can therefore conclude that groundwater recharge takes place during winter, which may confirm the relationship between a wet, cold climate and lake level fluctuations^23,26^.

**Table 1:** Geochemistry of surface water and groundwater from the Lake Urmia basin (Iran) and from the cored sedimentary sequences at the Shahr Chay River mouth (north-western area).

Field mission	Station	Type	Depth and comment on sampling	Latitude	Longitude	Altitude	Total alkalinity	Carbonate	Bicarbonate	C	T	pH	δ^18^O	δ^2^H	δ^13^C	^14^C analyse			Age ^14^C	σ
				N	E	(m)	(mg.L^-1^ CaCO_3_)	(mg.L^-1^ CO_3_^-^)	(mg.L^-1^ HCO_3_^-^)	(mS.cm^-1^)	(°C)		(‰ vs V-SMOW)	(‰ vs V-SMOW)	(‰ vs PDB)	Nr	F^14^C	1σ	(yr BP)	(yr)
Spring 2016	Golman 0	Well	Close to coring site	37°35′15.44"	45°15′19.70"	1278	184.0	0.0	224.5	1.178	14.8	7.35	− 8.6	− 57.1	− 9.42	E2468	0.9241	0.0025	635	20
	Golman 2	Coring	14.00	37°33′09,09"	45°16′31.20"	1270	520.0	0.0	634.4	219	29.2	5.8	3.3	− 7.7						
	Golman 3	Coring	14,2 m / pressurized sludge	37°33′09,09"	45°16′31.20"	1270	1140.0	0.0	1390.8	147.1	20.2	6.35	− 5.2	− 47.9	13.10	E2477	0.0157	0.0010	33,370	490
	Golman 4	Lake	0,60 m / at coring site	37°33′09,09"	45°16′31.20"	1270	564.0	148.8	385.5	207	21.3	8.02	− 3.9	− 33.7	1.14	E2476	0.9329	0.0025	560	20
	Shahrchay 1	River		37°31′35.24"	45°02′50.53"	1354	66.0	0.0	80.5	0.249	14	8.41	− 8.3	− 52.1	− 6.22	E2475	0.7488	0.0021	2325	25
	Shahrchay 2	River		37°33′16.93"	45°16′12.26"	1276	192.0	0.0	234.2	3.4	16.8	7.83								
	Dam Bridge 1	Lake	Surfaces / N side of the causeway	37°46′31.62"	45°19′47.04"	1268	976.0	0.0	1190.7	190.2	27.9	7.53	− 3.3	− 34.6	*9.00*					
	Hajilar 1	Well		37°33′22.00"	45°16′17.51"	1277	828.0	0.0	1010.2	3.12	15.4	6.29	− 10.2	− 66.1	14.20		0.0700	7.00		
	Hajilar 2a	Well		37°33′09.07"	45°16′08.95"	1276	698.0	0.0	851.6	12.22	15.7	6.12	− 10.5	− 69.7	19.11		0.0981	9.81		
	Hajilar 2b	Well		37°33′09.07"	45°16′08.95"	1276	414.0	0.0	505.1	12.39	16	6.63	− 10.9	− 71.0						
Autumn 2017	Kesh1	Well		37°32′28.788"	45°14′07.224"	1281		0.0	170.8	0.575	19.8	8.13	− 9.9	− 62.7	− 9.26	E2473	0.6914	0.0021	2965	25
															− 8.69	E2474	0.6923	0.0020	2955	25
	Bardesoor	River		37°26′14.592"	44°49′26.92"	1606		24.0	73.3	0.299	13.8	8.38	− 9.3	− 56.7	− 4.38	E2470	0.6798	0.0020	3100	25
	Saleh Abad	Well		37°31′16.626"	45°10′52.812"	1294		0.0	95.3	0.884	14.1	7.28	− 8.9	− 55.4	− 6.83	E2471	0.8525	0.0027	1280	25
	Golman 3	Coring	14.20	37°35′33.09"	45°16′31.20"	1268		0.0	3050.0	142	18.9	6.24	− 5.3	− 48.0	11.66	E2469	0.0206	0.0010	31,180	390
	Golman 5	Coring	5.25	37°35′33.86"	45°16′27.60"	1270		0.0	610.0	222	26.6	6.04	5.1	− 3.3	5.25	E2471	0.1604	0.0012	14,700	60
	Golman 6	Coring	8.00	37°33′28.854"	45°16′33.834"	1270		0.0	610.0	*206*	–	–	− 0.8	− 23.2	3.70	E2570	0.3000	0.0012	9670	30
	Golman 7	Coring	12,5 m / pressurized sludge	37°33′28.746"	45°16′33.75"	1270		–	–	–	–	–	− 0.8	− 23.2	3.70	E2570	0.3000	0.0012	9670	30

**Figure 4:**
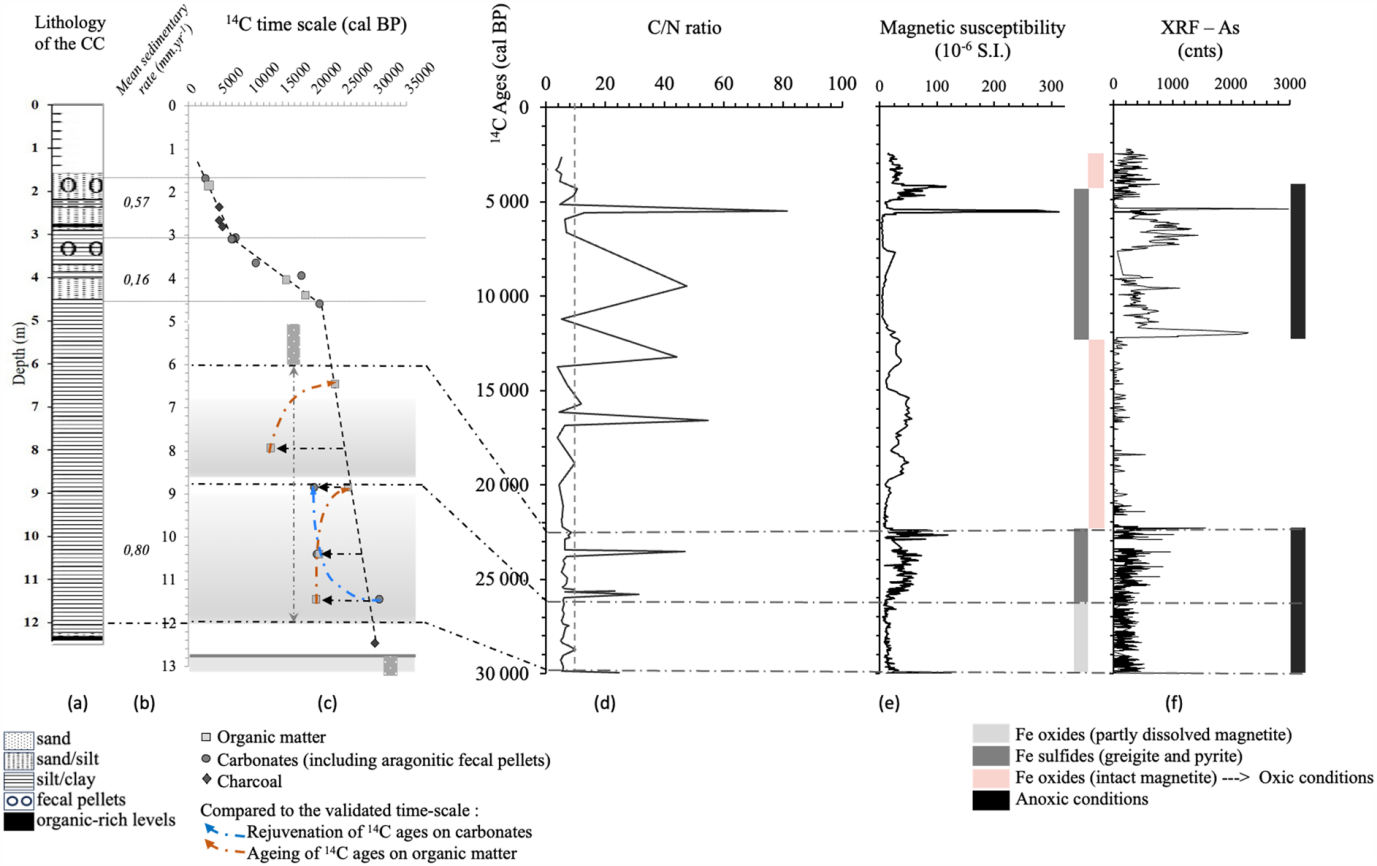
Lithology of the Composite core (**a**), mean sedimentary rates (**b**), the ^14^C timescale (**c**), and evolution of geochemical and sedimentological parameters against the ^14^C timescale: (**d**) C/N ratio of total organic matter, (**e**) magnetic susceptibility^21^, and (**f**) arsenic contents determined by XRF^23^.

See Figs. 2 and 4 in the main text for sample location.

The stable isotope contents of the G2-G5-G6 sludge and lake surface water samples define a clear evaporation line intercepting the LMWL at the point corresponding to the source of the Shahr Chay River samples, indicating that these waters and sludges originate via a single evaporation process from river water (Fig. 3A). However, the very similar isotopic data from lake surface waters near the coring site and north of the causeway do not confirm the impact of this process on water flow between the two sub-basins^10,24^. Curiously, the G3 data points did not lie on the evaporation line. However, as the G3 and G7 sludges emerged by artesianism from equivalent depths after passing through the same indurated sedimentary level, we assume the existence of the same evaporation process, defining an evaporation line crossing the G3 samples and intercepting the LMWL at the Hajilar boreholes. The significant H_2_S degassing from the Hajilar wells corresponds to that accompanying extraction of the G3 and G7 muds, confirming our assumption concerning the evaporation process.

Besides measuring δ^18^O/δ^2^H contents, we studied the carbon geochemistry in depth to constrain potential evolutions and imbalances that could affect the ^14^C chronologies of the lake sediment. The TDIC δ^13^C values and ^14^C activities were therefore determined in all water and sludge samples whenever their carbon content permitted (Table 1; Fig. 3B). The surface and groundwater in the Shahr Chay River catchment (excluding the Hajilar area) showed variable δ^13^C_TDIC_ (from − 6.8 to + 19.1 ‰ vs PDB) and A^14^C values (between 1.6 and 92.4 pMC), highlighting various processes such as the contribution of soil CO_2_, and the impact of limestone formations in the watershed. Although ^18^O and ^2^H contents of lake surface waters showed re-equilibration with the atmosphere coupled with the evaporation process (Fig. 3A), the corresponding ^14^C activity of 93.3 pMC does not indicate completion of this equilibrium with modern atmospheric CO_2_ (Fig. 3B,C). Geochemical interpretations are trickier for the muds and for the most enriched Hajilar groundwater. Although the δ^18^O-enriched values of the sludges indicate evaporation giving birth to lake brine formation (Fig. 2), their δ^13^C values were also highly enriched (+ 5.2 to + 13.1 ‰) and associated with relatively low ^14^C activities (1.6 to 16 pMC), pointing out specific geochemical processes and relatively old water. These geochemical processes appear to be related to groundwater from the Hajilar wells, whose isotopic contents (+ 19.1 ‰) are the most highly enriched ^13^C contents of the entire basin.

Considering all these stable isotope data, the muddy samples recovered from the drillings can be interpreted as brines resulting from a strong evaporation of the Lake Urmia surface water and then trapped in the sediments. The first step of this trapping process was observed during the fieldwork when the already salty waters of the lake at the coring site in 2016 underwent an intense evaporation phase that produced in the following years thick salt crust, later covered by a thin layer of ochre-colored aeolian dust (Fig. 1E). We can therefore consider that the brines embedded in the sediments of Lake Urmia are remnants of highly evaporated lake water (equivalent to that of present-day Lake Urmia—see Table 1), formed and trapped during arid climatic conditions associated with significant aeolian input. This formation of lenses may have occurred very regularly over time, since environmental reconstructions of Lake Urmia have shown that the lake has always been more or less salty^21–23^.

To avoid biases related to pH variability in the water-carbon system, δ^13^C values of theoretical CO_2_ in equilibrium with TDIC were calculated using the constants and fractionation factors of the dissolved carbon species involved^27^. Two end-members were defined, the first referring to soil P_CO2_ related to current vegetation (average δ^13^C value of − 17‰^23^ and 100-pMC ^14^C activity), the second representing the generally accepted average composition of geological marine limestones, assumed to 0 ‰ and 0 pMC for δ^13^C and A^14^C respectively. The graph of calculated δ^13^C_theoretical CO2_ vs A^14^C highlights several geochemical processes given the endmembers defined above (Fig. 3C): (1) process 1. reflects the rebalancing of the lake's surface waters with atmospheric CO_2_, although river input to the lake produced a ~ 35 pMC aging at the time of coring, (2) process 2. corresponds to mixing of the two members "soil" and "^14^C-free limestone", inducing ^13^C enrichment of equilibrium CO_2_ and decrease in ^14^C activities of surface waters, (3) evolution 3. emphasizes the unique contribution of soil CO_2_ for the Shahr Chay River and groundwater, associated with radioactive decay, and (4) process 4. is particularly remarkable, highlighting a very significant ^13^C enrichment of sludge TDIC, associated with a slight evolution in their A^14^C, in relation with early diagenesis (sulfate-reduction) of organic matter; bacterial release of oxygen from iron oxides leads to the production of iron sulfides, such as greigite and pyrite as found in Lake Urmia deposits (Fig. 4)^21,23^. Although this has only been demonstrated in freshwater lakes^28^, process 4. also appears to significantly increase the secondary formation of ^14^CO_2_ in salt lake deposits and thus modify ^14^C activities, which will have an impact on both the understanding of water fluxes and the validation of sediment ages. Hajilar groundwater is consistent with Process 4, confirming its common origin with the brines reached in Core G3 (Fig. 3B,C).

In summary, the clear logarithmic curve (δ^13^C = -2.91 * ln[A^14^C] + 13.95) shown in Fig. 3B and the four geochemical processes highlighted in Fig. 3C clearly characterize the main geochemical end-members of our system, namely G3 sludge and lake surface water. Only the Hajilar wells appear to be connected to the brines, as confirmed by the regular deepening of these wells by the local population in order to reach fresh water, as the pumped water becomes increasingly saline. River water and other wells show no relationship with the sludge, indicating confined brackish environments. Although the aquifers present a piezometric decline on a regional level^29^, the groundwater in the riparian zone of Lake Urmia to the west therefore does not appear to be directly connected to the lake, except for the shallow aquifer linked to the base flow of the Shahr Chay River, itself mainly fed by winter precipitation and spring snowmelt from the Zagros Mountains, as indicated by isotopic contents of both spring and autumn river samples plotted close to winter precipitation and far away from summer ones over the region (Fig. 3A).

## ^14^C chronology of Lake Urmia lacustrine sediments

Although very complex at first glance, the radiocarbon time scale of Lake Urmia composite core was established and validated in close relation to the modern hydrogeochemical system of the lake: 16 of the 24 AMS-^14^C dates were determined on authigenic charcoal, inorganic carbonates and diffuse organic matter, after correction of geochemical processes affecting these based on previously acquired geochemical data^21–23^. These dates establish a reliable time scale for the Lake Urmia C-core (dotted line; Fig. 2c). However, the eight remaining ^14^C ages at the C-core base show quite significant discrepancies between carbonate and diffuse organic fraction dates determined at the same levels (Fig. 2; Table 2, Methodology), all of which point to rejuvenation of the organic and carbonate dates compared with the validated time-scale. As an example, in the 12–9 m section, these clearly visible rejuvenations are opposite in direction: (1) at 11.45 m deep, the organic matter appears much younger than the expected age with a maximum ca. 9.0 kcal-^14^C difference, and it then evolves to equilibrium (true ages) towards the validated time scale (brown arrow), whereas (2) carbonate datings are close to the certified chronology at 11.45 m deep but evolve towards younger and younger ages (blue arrow) that produce a more pronounced gap with respect to the validated time scale as sedimentation proceeds (maximum ca. 4.5 kcal-^14^C difference at 8.85 m deep).

**Table 2:** Radiocarbon dating on carbonated and organic samples from the Lake Urmia composite core, including correction steps taking into account the detrital fractions and the 2000-yr hard water effect^21–23^.

Sample	Depth (cm)	Type^(a)^	Sample Nr	F^14^C(pMC)	σ^(b)^	Measured^14^C ages	σ^(b)^	^14^C Ages corrected from detrital fraction	^14^C Ages corrected from a 2-kyr HWE	Calibrated ^14^C ages	
								(yr BP)	(yr BP)	(cal BP)	2σ
Golman-5 core											
G5	2.34	Charcoal	B2087	0.5824	0.20	4343	25	4340	4340	**4910**	− 60/ + 60
G5	2.66	Charcoal	B2088	0.5787	0.20	4394	22	4390	4390	**4940**	− 75/ + 100
Composite core (Golman-6 and Golman-7 cores)											
G7-S1	1.70	CaCO_3_	A17861	0.5580	0.320	4265	26	4560	2560	**2720**	− 205/ + 30
G7-S1	1.85	OM	B2295	0.5306	0.002	5090	25	3090	3090	**3295**	− 75/ + 65
G7-S2	2.80	Charcoal	B2097	0.5547	0.400	4735	22	4730	4730	**5475**	− 145/ + 105
G7-S2	3.07	CaCO_3_	62.008	0.3334	0.420	8825	34	8730	6730	7595	− 80/ + 70
G7-S2	3.09	Black FP ^(^**^)^	A21543	0.3880	0.0036	7600	80	(Secondary carbonate precipitation suspected)			
G7-S2	3.09	White FP ^(^**^)^	A21542	0.3666	0.0035	8060	80	8060	6060	**6920**	− 180/ + 240
G7-S2	3.09	CaCO_3_	62.006	0.3579	0.410	8300	33	8300	6300	**7215**	− 55/ + 90
G7-S3	3.65	CaCO_3_	62.007	0.2377	0.530	11,540	42	11,520	9520	**10,850**	− 245/ + 225
G7-S3	3.95	CaCO_3_	62.002	0.1138	0.12	17,450	90	16,870	14,870		
G7-S3	4.05	OM	B2297	0.1529	0.100	15,090	66	15,090	13,090	**15,695**	− 230/ + 235
G7-S3	4.15	OM	B2100	0.0569	0.59	23,032	47	23,030	21,030		
G6-S2	4.40	OM	B2304	0.1124	0.0011	17,560	76	17,560	15,560	**18,845**	− 135/ + 135
G6-S2	4.60	CaCO_3_	62.001	0.0662	0.120	21,810	140	19,470	17,470	**21,115**	− 385/ + 575
G6-S4	6.45	OM	B2300	0.0689	0.0005	21,490	63	19,490	17,490	**23,510**	− 260/ + 255
G6-S5	7.95	OM	B2299	0.1913	0.003	13,286	114	13,290	11,290		
G7-S6b	8.85	CaCO_3_	62.003	0.0554	0.12	23,238	170	18,700	16,700		
G7 S6b	8.85	OM	B2099	0.0830	0.123	23,240	82	23,240	21,240	**25,610**	− 555/ + 375
G7-S7	10.40	CaCO_3_	62.004	0.0551	0.11	23,280	170	19,050	17,050		
G7-S7	10.40	OM	B2104	0.0889	0.71	19,442	57	19,440	17,440		
G7-S8	11.45	OM	B2101	0.0942	0.68	18,976	45	18,980	16,980		
G7-S8	11.45	CaCO_3_	62.005	0.2139	0.10	30,890	390	28,500	26,500		
G7-S8	12.46	Charcoal	B2301	0.0005	0.001	25,590	96	25,590	25,590	**29,950**	− 290/ + 165

The timescale is expressed in calendar ages. See Figs. 2 and 4 in the main text for sample location.

(*)Apparent Hard Water Effect (HWE).

(a)CaCO_3_: total carbonate fraction; OM: diffused organic matter; FP: fecal pellets (*Artemia*).

(b)Error bars represent one sigma deviation

(c)Accuracies on ^13^C measurements are of ±0.05 and ±0.02 ‰ vs V-PDB for carbonates and organic matter respectively

(d)References: - Reimer P.J. *et al.* The IntCal20 Northern Hemisphere Radiocarbon Age Calibration Curve (0–55 cal kBP).* Radiocarbon* 62(4), IntCal20: Calibration Issue (2020); Stuiver, M., and Reimer, P.J., Reimer, RW, 2021. CALIB Radiocarbon Calibration, URL: http://calib.

Certified ^14^C dating are in [bold].

In salt lakes, several biogeochemical mechanisms can result in reverse rejuvenation by interacting with the carbon cycle. Redox reactions play a major role at the interfaces of underlying aquifers, due to the particularly steep gradient between saline and freshwater^30^. Mobilization of sulphur (and consequently H_2_S), and formation of CH_4_ and secondary CO_2_ can interact with C-O components at both sediment–water and sediment-groundwater interfaces at various depths^30^. Several mechanisms can lead to CH_4_ production/interaction/degradation in the lake^28,30–37^: (1) hydrogenotrophic methanogenesis, the main CH_4_ production pathway in natural environments, (2) acetoclastic methanogenesis, mainly observed in cold, temperate freshwater ecosystems, and (3) methanotrophy by oxidation of organic matter under oxic conditions, in anaerobic sulfate- or nitrogen-rich environments, in marine environments, or linked to denitrification and significant CO_2_ formation. Methanotrophic degradation of organic matter can therefore occur in Lake Urmia sediments as a result of several concomitant, albeit extremely complex, processes in relation with the hydro-sedimentary dynamics of the Shahr Chay river basin and its delta, these end-of-stream zones being considered as hot spots for CH_4_ in relation with the generally high rates of deposited organic matter^28,38^. This process represents the predominant mode of CH_4_ production in hypersaline environments^39^, although the most recent studies described aerobic methanogenesis in relation to environmental factors such as sediment mineralogical composition, O_2_ concentration, pH, salinity or temperature^39,40^, but also with hydro-environmental factors such as precipitation or the absence of precipitation^41^ or with oxygenated spells due to fluctuations in groundwater level^39^.

The net depletion of δ^13^C_TDIC_ values from the group gathering deep groundwater and sludges (+ 19.1‰) to the Lake Urmia surface water (+ 1.1‰; Fig. 3B) is identical to that observed along the highly reactive hydrogenotrophic methanogenesis pathway in natural environments^32,39^, whether in alkaline lakes^35,42^ or deep freshwater reservoirs^43^. However, the range of isotopic values in Lake Urmia differs greatly from those in high-latitude lakes^35,42^, probably due to its high salinity or the composition of its organic primary production. The main phases of methane formation in Lake Urmia sediments coincide with the 12–6 m deep section of the C-core and correspond to a high sedimentation rate, anoxic conditions, and the low C/N ratio underlining methanogenetic processes^23,44^ (Fig. 4d).

Changes in ^14^C ages can also occur through CO_2_ production from methanotrophic oxidation of organic matter. The high ^13^C_TDIC_ enrichment of groundwater and sludge (Fig. 3B) does not seem compatible with an anaerobic methane oxidation process, which normally produces very low groundwater δ^13^C_TDIC_ values^31^. However, the presence of sulfides (greigite and pyrite) and Fe-oxides (magnetite) in the Lake Urmia sequence, which indicate both aerobic and anaerobic methane oxidation processes, could easily explain the alternation of oxic and anoxic sedimentary conditions in this salt lake^21,23^ (Fig. 4e,f) and thus be compatible with the very high δ^13^C_TDIC_ values in deep groundwater and muds (Hajilar groundwater and G3 sludges).

We attempted to calculate the percentage of CO_2_ that, potentially produced by methanotrophy, would cause the observed rejuvenation of C-core ^14^C ages. Assuming isotopic equilibrium and constant reservoir composition, our calculations were based on a Rayleigh process considering ^13^C contents and ^14^C activities of initial and final CO_2_ in the system (cf. Methodology). The ^13^C isotopic contents of CO_2_ after methane production increase relative to initial values. The production of a CH_4_ fraction between 0.25% and 0.30% in brines trapped in lake sediments would result in a ^14^C enrichment of 4.6 to 5.7 pMC in the residual CO_2_, values of the same order of magnitude as in our data.

These results led us to explore the possible origin of the rejuvenation of ^14^C ages in the C-Core through a newly formed younger carbonate fraction. As Lake Urmia is located in a highly tectonic zone, containing petroleum sources, the methane revealed could be of deep-seated origin, rising via basin-wide porosity and then captured in highly saline sedimentary levels or porous media. However, such deep-seated methane would give rise to ^14^C-free secondary carbonates, contradicting our observations of rejuvenation rather than aging of the total carbonate fraction. The secondary carbonate fraction might also result from CO_2_ production via early diagenesis of organic matter by sulfate-reducing bacteria, or direct anaerobic methane oxidation and secondary, more recent ^14^CO_2_ formation^45,46^. This bacterial release of oxygen from iron oxides would lead to the production of iron sulfides (greigite -Fe_3_S_4_- and pyrite -FeS_2_-) from magnetite (Fe_3_O_4_), as observed in Lake Urmia^21,22^. Our data confirm this in-situ CH_4_ and CO_2_ production, particularly in the 12–9 m depth range (Fig. 4). This is consistent with the rejuvenation of the samples, which decreases upwards, indicating oxygen depletion and hence reduced secondary carbonate precipitation. The presence of arsenic sulfide and H_2_S confirms this early diagenesis, well advanced at the depths considered.

## Discussion

**Figure 5:**
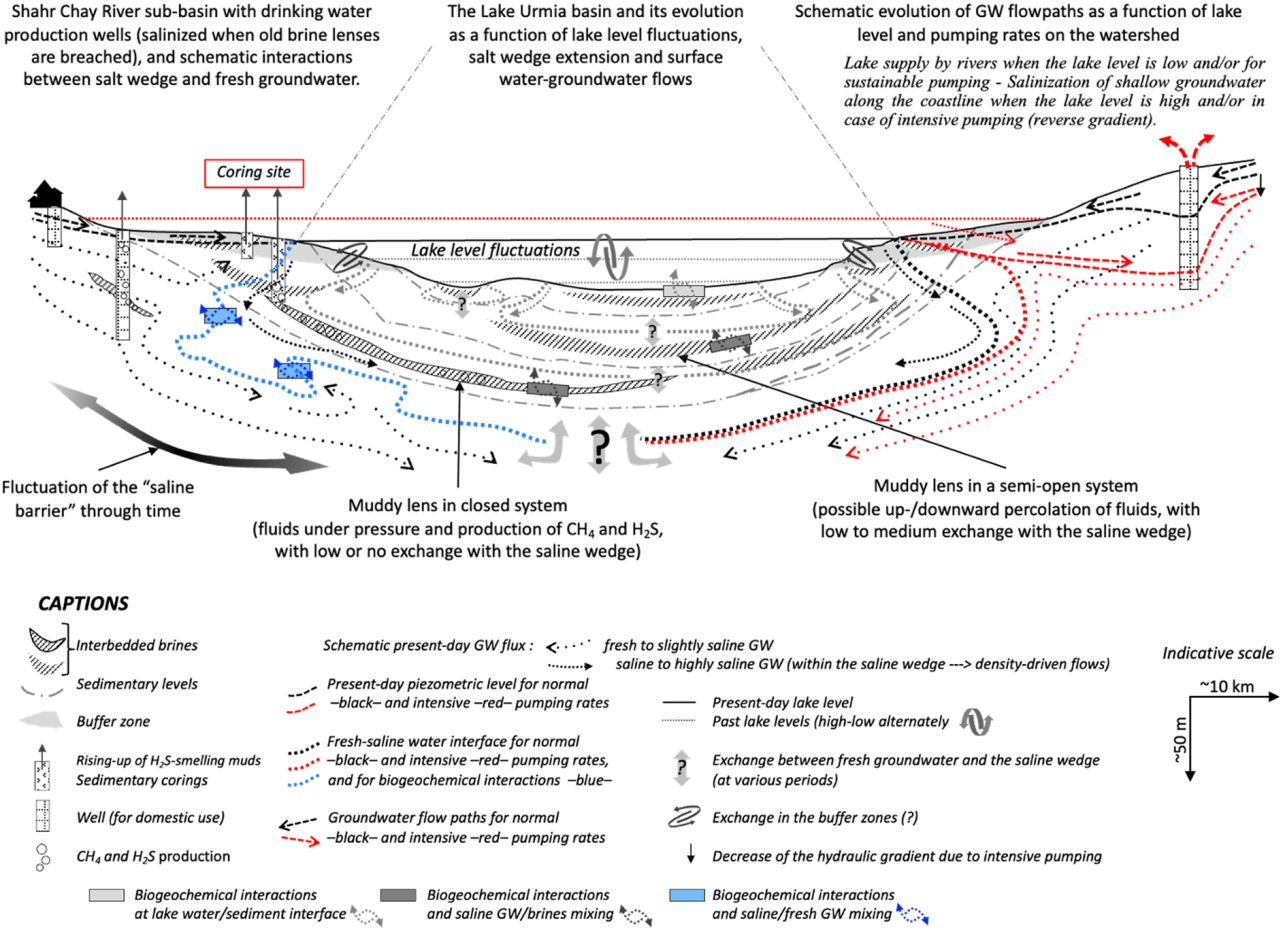
Schematic representation of the overall exchange between Lake Urmia, surface water and groundwater in the Shahr Chay River sub-basin, including the behavior of lake sediments that form a near-tight barrier to direct groundwater recharge.

All these characteristics make Lake Urmia a unique example of specific sedimentary alternation of trapped brines associated with greenhouse gas (GHG) production, and gentle sedimentation in the absence of CH_4_ or secondary CO_2_ effects, leading us to picture lake dynamics in terms of sediment-surface water-groundwater relationships (as represented in the conceptual schematic Fig. 5). The present study therefore points out that methane originates in brine lenses formed and trapped in the sediments of Lake Urmia, mainly during arid climatic spells associated with a high aeolian input. Such spells have been already recognized from our previous works before ca 30 cal kBP and between ca 29 and 20 cal kBP^21,23^. This has been also observed in 2016–2017, when the already extremely salty mud resulting from intense evaporation partially solidified and was covered by a thin layer of ochre aeolian dust (Fig. 1). This formation of brine lenses at the sediment–water interface probably occurred very regularly during anoxic phases, constituting an almost impermeable barrier to exchanges with the water table on the banks or at the bottom of the lake. We also confirm the existence of a saline wedge in the lake bottom sediments, constituting a quasi-impermeable barrier to exchanges between the aquifers and the surrounding lacustrine deposits (Fig. 5). Although it is difficult to extend our interpretations to the whole of the lake basin (complex upscaling due to highly heterogeneous geological features and hydrological interactions) our results show that, apart from the shallow groundwater linked to river baseflow, groundwater does not support the lake level, being strongly impacted by this saline wedge. The very low hydraulic gradient between the lake and surrounding aquifers^16,47,48^ may affect the time required for the saltwater-freshwater interface to stabilize after a change in recharge, and therefore the dynamics of successive salt intrusions over time^5,15,18,49^. With this temporal difference in basin response, groundwater diverted by the salt wedge flows beneath the lake in the regionally predominant south-north direction^16^. Without a reduction in anthropogenic pressure on surface waters supported by sustainable economic and societal strategies^45,50^, Lake Urmia will not quickly regain a sufficient recharge from incoming rivers and will therefore be unable to safeguard ecosystems.

Our study of Lake Urmia clearly shows that regularly trapped brines in lake sediments are both markers of short-term regressive phases within the globally high lake level period of the Late Pleistocene, and exceptional laboratories for understanding geochemical evolution in closed environments, with dissolution-recrystallisation processes strongly disrupting radiocarbon chronologies in relation to the impact of biological methane production within the deposits and associated reactions within the tightly-linked carbon system. Methane emissions from lake ecosystems, as suggested by global modelling, are increasingly recognized owing to better understanding of the processes involved and the effect of rising temperatures^46^, as well as to the study of saline lakes such as Lake Urmia, which can contribute significantly to these emissions due to their shallow depth, precluding methane absorption in the water column. Our study, which highlighted the processes of methanogenesis, could be followed up by more quantitative work using 3D mapping of the lake and its sediments, as well as geochemical analyses and modelling of the methane fluxes produced, in relation to lacustrine high- or lowstands.

We conclude that, although uncertainties remain regarding their contribution to both global CO_2_ and CH_4_ budgets^51,52^, estimating the contribution of saline lakes to GHG rates is crucial. Thorough understanding of CH_4_ production processes and the spatiotemporal functioning of endorheic or slightly saline lake basins, i.e., before aridification-related salinization leads to "salt wedge" functioning, represents a challenge for the development of appropriate mitigation^53,54^, considering GHG source reduction, ecosystem recovery and resilient socio-economic strategies.

## Methodology

### Study site: core description and sampling

In 2016 and 2017, several sedimentary sequences were cored in the SW part of Lake Urmia basin, from the very recently dried out, although muddy, part of the lake (Tudryn et al. 2021). Among the 8 cores collected with a mechanic corer, two were subjected to an in-depth multi-parameter sediment study, namely the Golman 7 and Golman 6 cores extending to depths of 12.5 m and 8 m, respectively (Tudryn et al. 2021; Kong et al. 2022a and 2022b). These two cores allow constitution of a homogenous lacustrine composite core based mainly on magnetic susceptibility. Only the depth of 1.6-m and above is missing at the core top.

## Precipitation

In order to define the local Meteoric Water Line (LMWL) corresponding to rainwater on the Shahr Chay River basin, data from the GNIP database (IAEA/WMO GNIP Network, 2022) were used for the three main stations surrounding Lake Urmia: Diyarbakir and Dalbahce (Turkey) and Tehran (Iran). See Fig. 3 for station location.

### Stable isotopes

Stable oxygen and deuterium (δ^18^O, δ^2^H) contents, expressed as percentages versus V-SMOW (Vienna Mean Ocean Water) values obtained on the TDIC (Total Dissolved Inorganic Carbon) for water samples from both surface and groundwater (lake, wells and rivers), as well as for mud samples rising up from the coring tubing. ^18^O and ^2^H contents were measured on a laser spectrometer at the GEOPS Laboratory (Orsay, France). Analytical uncertainties, including laboratory errors, were ± 0.1‰ and ± 2.0 ‰ for δ^18^O and δ^2^H, respectively.

Stable carbon isotope contents (δ^13^C) of Total Dissolved Inorganic Carbon (TDIC), expressed as percentages versus V-PDB (Vienna Pee Dee Belemnite standard) were measured on a VG SIRA 9 mass spectrometer at the LOCEAN laboratory (Paris, France) and calibrated with respect to the NBS19 calcite standard. Analytical uncertainties, including laboratory errors, were ± 0.1‰ for δ^13^C.

### Methane calculation

The equations defining the isotopic composition of dissolved CO_2_ during methanization are as follows:

$${{\updelta}}^{{{13}}} {\text{C}}_{{\text{remaining CO2}}} ={{\updelta}}^{{{13}}} {\text{C}}_{{\left( {{\text{CO2}}} \right)0}} - {{\upvarepsilon}}*{\text{ ln}}\left( f \right)$$

$${\text{A}}^{{{14}}} {\text{C}}_{{\text{remaining CO2}}} = {\text{A}}^{{{14}}} {\text{C}}_{{\left( {{\text{CO2}}} \right)0}} - {{\upvarepsilon}}* \, 0,{23 }*{\text{ ln}}\left( f \right)$$

With: ε = (a*10^6^/T_K_^2^) + (b*10^3^/T_K_) + c = 10^3^.ln(α_CO2-CH4_) and ^14^C variation (%) = 0.23 * ε

**Table:** 

T	T_K_	Fractionation factors^#^			ε(Enrichment factor)^##^	^14^C variation (%)^###^
(°C)	(K)	a	b	c		
20	293.15	2.2800	15.176	− 8.38	69.91981	16.08156

The values are − 17‰ for the δ^13^C of initial CO_2_, far exceeding that of CH_4_ by − 80‰, and 100 pMC for the ^14^C activity of final CO_2_, a higher activity than that assumed for CH_4_, which is 84 pMC.

**Table:** 

Original conditions		*f* *	Final conditions	
A^14^C_(CO2)0_	δ^13^C_(CO2)0_		δ^13^C_remaining CO2_	A^14^C_remaining CO2_
90	− 17	1.00	− 17.0	90.0
90	− 17	0.95	− 13.4	90.8
90	− 17	0.90	− 9.6	91.7
90	− 17	0.85	− 5.6	92.6
90	− 17	0.80	− 1.4	93.6
90	− 17	0.75	3.1	94.6
90	− 17	0.70	7.9	95.7
90	− 17	0.65	13.1	96.9
90	− 17	0.60	18.7	98.2
90	− 17	0.55	24.8	99.6

^#^According to Bottinga (1969) for T values ranging from 0 to 700 °C;

^##^According to Saliège, JF, Fontes, JC. Essai de détermination expérimentale du fractionnement des isotopes ^13^C et ^14^C du carbone au cours de processus naturels. *International Journal of Applied Radiation and Isotopes* **35**(1), 55–62 (1984).

^###^According to Aravena R., et al*.* Distribution and isotopic characterization of methane in a confined aquifer in southern Ontario, Canada. *Journal of Hydrology* **173**, 51–70 (1995).

^*^f = remaining CO_2_ fraction after methanation.

### Radiocarbon dates

^14^C dating was performed at the ECHoMICADAS facility (CNRS-CEA Saclay, France). AMS-^14^C measurements were performed on the inorganic carbonate fraction and diffused organic matter of bulk samples from the C-core, on handpicked plant remains and charcoals when present, as well as on water and muddy coring samples.

The organic fraction was subjected to the standard chemical protocol for AMS analyses, namely three successive hydrochloric acid/sodium hydroxide/hydrochloric acid baths, rinsing with deionized water up to neutral pH, and gently drying at 60 °C overnight.

CO_2_ gas was obtained (1) for organic samples by burning these at 860 °C for 30 min, under vacuum, in the presence of a mixture of copper (II)-oxide/copper (III)-oxide and silver thread, and (2) for carbonates, by H_3_PO_4_ acid attack under vacuum for one night in a thermostatic bath, and (3) for water and muddy samples, by direct H_3_PO_4_ acid attack on the TDIC of the samples.

AMS-^14^C targets were then obtained by graphitization of the CO_2_ gas on powdered iron with hydrogen at 650 °C for 100 min, and graphite compression under analytical plots. Aliquots of the CO_2_ gas were then used for associated ^13^C measurement. Graphite sources were prepared in the GEOPS laboratory, and counted by accelerator mass spectrometry at LSCE laboratory (ECHoMICADAS facility, France).

As regards A_o_ activity (initial activity), each dating of present-day surface water or groundwater refers to the ^14^C activity of the atmospheric CO_2_ at the time of sampling, i.e., 2016–2017, assumed to 101.8 pMC. (Hua Q. et al*.* Atmospheric radiocarbon for the period 1950–2019. Radiocarbon, 10.1017/RDC.2021.95 (2021)).

Analytical uncertainties, including laboratory errors, were ± 0.1‰ for δ^13^C and from 0.5 to 0.8 pMC for ^14^C activity. All dates established on sediments were converted to calendar ages according to the revised calibration program IntCal20 (Execute Version 1.10 html 2020; Table 2; Reimer et al., 2020).

## Data Availability

The datasets generated and/or analyzed during the current study are not publicly available due to a 2-year embargo imposed on the production and recording of data, but are available from the corresponding author on reasonable request.
