# Author Correction: Salt wedges and trapped brines of low-latitude endoreic saline lakes as potential modulators of GHG emission

**DOI:** 10.1038/s41598-024-62197-7

**Published:** 2024-05-27

**Authors:** Elisabeth Gibert-Brunet, Alina Tudryn, Ting Kong, Piotr Tucholka, Seyed-Hani Motavalli-Anbaran, Christelle Marlin, Aurélie Noret, Mohammad Lankarani, Hesam Ahmady-Birgani, Gilda Karimi

**Affiliations:** 1 CNRS, UMR 8148-GEOPS, University of Paris-Saclay, 91405 Orsay, France; 2 Faculty of Geology, Warsaw University, 02-089 Warsaw, Poland; 3 Institute of Geophysics, University of Tehran, Tehran, Iran; 4 School of Geology, University-College of Science, University of Tehran, Tehran, Iran; 5 Faculty of Natural Resources, Urmia University, Urmia, Iran; 6 Faculty of Biological Sciences, Kharazmi University, Tehran, Iran

Correction to: *Scientific Reports* 10.1038/s41598-023-48148-8, published online 30 November 2023

In the original version of this Article Affiliation 5 was incorrectly given as ‘Faculty of Natural Resources, University of Urmia, Urmia, Iran’. The correct affiliation is listed below.

Faculty of Natural Resources, Urmia University, Urmia, Iran.

The original Article has been corrected.

